# Thermomechanical Behavior of Molded Metallic Glass Nanowires

**DOI:** 10.1038/srep19530

**Published:** 2016-01-20

**Authors:** Daniel J. Magagnosc, Wen Chen, Golden Kumar, Jan Schroers, Daniel S. Gianola

**Affiliations:** 1 Department of Materials Science and Engineering, University of Pennsylvania, Philadelphia, Pennsylvania 19104, USA; 2 Department of Mechanical Engineering and Materials Science, Yale University, New Haven, Connecticut 06511, USA; 3 Department of Mechanical Engineering, Texas Tech University, Lubbock, Texas 79409, USA; 4 Department of Materials, University of California, Santa Barbara, California 93106, USA

## Abstract

Metallic glasses are disordered materials that offer the unique ability to perform thermoplastic forming operations at low thermal budget while preserving excellent mechanical properties such as high strength, large elastic strain limits, and wear resistance owing to the metallic nature of bonding and lack of internal defects. Interest in molding micro- and nanoscale metallic glass objects is driven by the promise of robust and high performance micro- and nanoelectromechanical systems and miniature energy conversion devices. Yet accurate and efficient processing of these materials hinges on a robust understanding of their thermomechanical behavior. Here, we combine large-scale thermoplastic tensile deformation of collections of Pt-based amorphous nanowires with quantitative thermomechanical studies of individual nanowires in creep-like conditions to demonstrate that superplastic-like flow persists to small length scales. Systematic studies as a function of temperature, strain-rate, and applied stress reveal the transition from Newtonian to non-Newtonian flow to be ubiquitous across the investigated length scales. However, we provide evidence that nanoscale specimens sustain greater free volume generation at elevated temperatures resulting in a flow transition at higher strain-rates than their bulk counterparts. Our results provide guidance for the design of thermoplastic processing methods and methods for verifying the flow response at the nanoscale.

Metallic glasses (MGs) are a unique class of materials with a suite of attractive properties resulting from the combination of metallic bonding character with an amorphous structure^1^. Interest in MGs has been driven by their many advantageous mechanical properties as compared to crystalline metals. Large elastic strain limits, high strength, potentially high fracture toughness, and good wear resistance are among the excellent mechanical properties exhibited by MGs^2^,^3^,^4^,^5^. In addition, above the glass transition temperature (*T*_*g*_), MGs soften in the supercooled liquid region allowing for facile processing through thermoplastic forming operations^6^. Taken together, the excellent mechanical properties, facile processing, and lack of intrinsic internal length scale (i.e. grain size) make MGs appealing for use in structural applications especially at the micro and nanoscale such as in micro- and nanoelectromechanical systems (MEMS and NEMS) devices^7^,^8^,^9^,^10^,^11^,^12^.

Production of nanoscale features from bulk specimens has been demonstrated via embossing^6^,^13^ and tensile drawing^14^,^15^. However, the role of dimensional confinement and reduced length scales has been shown to affect the thermomechanical response of nanoscale glasses, albeit with conflicting results^16^,^17^,^18^,^19^,^20^. An understanding of the nanoscale plastic deformation needed to perform precise and controlled thermoplastic molding of micro- and nanoscale MG devices thus necessitates studies of size dependent flow at elevated temperatures. Despite several recent reports of length-scale mediated transitions in plastic behavior^21^,^22^,^23^,^24^, the vast majority of these small-scale mechanical experiments have been performed at room temperature (i.e. well below *T*_*g*_).

The flow behavior of bulk MGs in the supercooled liquid region (above *T*_*g*_), on the other hand, has been investigated extensively through creep studies^25^,^26^, uniaxial tension or compression^27^,^28^,^29^,^30^, and nanoindentation^31^, and provide a good foundation before examining potential influences from reduced dimensionality. Such studies have produced a generalized deformation map predicting the onset of flow in MGs at ~0.7*T*_*g*_ and at applied stresses orders of magnitude less than the room temperature shear strength. In this flow regime the deformation mode (Newtonian or non-Newtonian flow) and failure morphology depend on the applied temperature, strain-rate and loading method^30^. As the strain-rate is increased the flow changes from Newtonian to non-Newtonian, as indicated by a stress overshoot during initial yield, strain-rate sensitivities 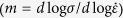 less than 1, and increased shear character in the failure morphology^28^,^30^. While the origins of the transition to non-Newtonian flow remain unclear, many observations are well explained through a free volume model in which the temperature dependent free volume determines the viscosity^28^,^32^,^33^. In this framework, the Newtonian to non-Newtonian transition is dictated by a competition between free volume production and annihilation during plastic deformation and thermal relaxation, respectively^33^.

While bulk MG flow behavior has been extensively investigated, comparatively little is understood about elevated temperature deformation at small length scales despite strong interest in employing thermoplastic deformation at the nanoscale. In order to advance the understanding of high temperature deformation of MGs at the nanoscale we performed novel thermomechanical experiments on Pt-based MG nanowires produced by thermoplastic molding^6^. First, large arrays of nanowires were produced by hot-pulling well above the glass transition temperature. Under appropriate conditions, hot-pulling induced significant homogeneous tensile elongation and reduction in the diameter of MG nanowires across large areas. To provide a clearer picture of this process we performed thermomechanical tensile experiments on individual molded MG nanowires at temperatures near *T*_*g*_ and examined the flow behavior and failure morphology across a range of viscosities. We further compared these results to thermomechanical compression experiments on bulk specimens of the same composition. Our experiments provide quantitative thermomechanical properties based on individual nanowire experiments, which provide crucial insight for further developments in nanoscale thermoplastic processing.

## Results

**Figure 1:**
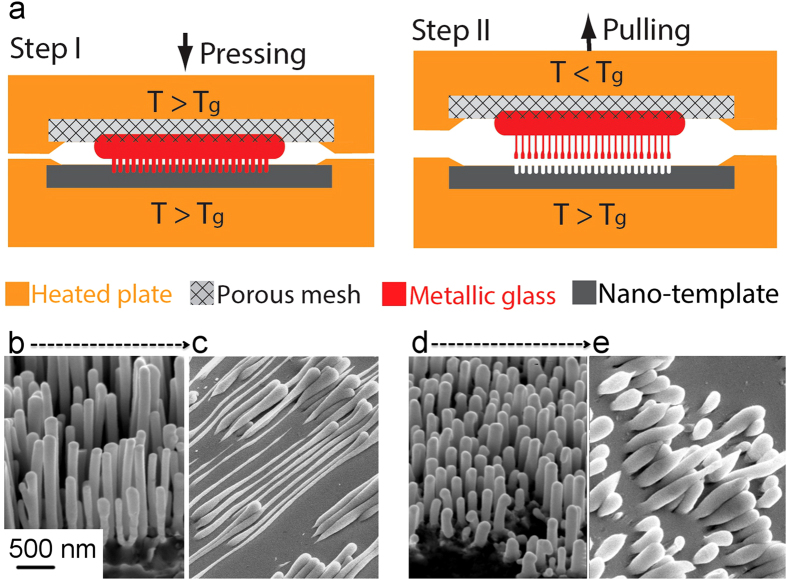
(**a**) Illustration of hot-drawing process for fabrication of multiple metallic glass nano-wires. During step I, the metallic glass fills the micro-porous mesh and nano-template. In step II, the metallic glass nano-wires are drawn out of nano-template by introducing a temperature mismatch between the top and the bottom heating plates. (**b**–**e**) Show example hot-pulling morphologies. (**b**,**c**) Show initially long (1.50 μm lengths) before and after hot-pulling respectively. (**d**,**e**) Show initially short (0.45 μm lengths) before and after hot-pulling respectively. Generally, the initially long nanowires deform to a greater extent than initially short wires as evidence by the difference between (**c**,**e**).

Large scale hot-pulling (*T*_*g*_<*T*<*1.07T*_*g*_) of nanowire arrays, illustrated in Fig. 1a, resulted in copious superplastic deformation. Depending on the initial dimensions of the molded nanowires (Fig. 1b,d) the deformation morphology changed as summarized in Table 1. Initially long nanowires (Fig. 1b) with lengths of 1.50 μm showed extensive deformation and the diameters of some nanowires were uniformly reduced from 150 to 50 nm (Fig. 1c). In addition, when the hot-pulled wires were pulled until failure, the nanowire plastically deformed down to a sharp point. Such uniform deformation is indicative of Newtonian deformation at low strain-rates. In contrast, initially short nanowires (Fig. 1d) with lengths of 0.45 μm exhibited very little elongation and an early onset of necking leading to sharp tips (Fig. 1e). While the true strain-rate in these array experiments is unknown, these observations suggest that reducing the initial length increases the applied strain-rate by approximately a factor of three resulting in an apparent transition to non-Newtonian flow. It is important to note that the rounding and apparent increase in diameter from Fig. 1d–e is driven by surface tension after separating from the mold. These qualitative observations motivated us to further investigate the transition between flow regimes and quantify the flow behavior by performing quantitative thermomechanical experiments on individual molded nanowires.

**Figure 2:**
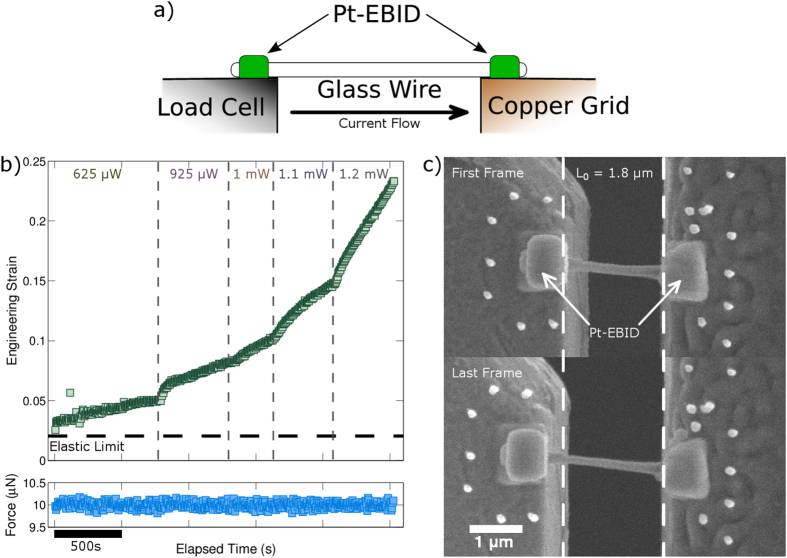
A schematic of the testing configuration is shown in (**a**) where a nanowire is gripped between the load cell and a TEM half grid with Pt-based EBID. An electrical current can then be passed from the load cell to the half grid. An example raw data set is shown in (**b**), which includes strain measured from the image sequence and force over time. Regions of different power dissipation are also indicated. To emphasize the large strains achieved through thermoplastic deformation the first and final frame of the testing sequence are shown in (**c**) with the original length indicated by the dashed lines.

To measure the thermomechanical properties of individual nanowires in creep-like conditions, stress and strain must be applied and measured, respectively, under a variety of temperatures. Here, the temperature is increased by passing an electrical current through the nanowire during mechanical testing, giving rise to Joule heating in the nanowire; the testing configuration is shown schematically in Fig. 2a. The temperature is controlled by the power dissipation (see Supplementary Materials for additional details). For an in depth description of the experimental setup and analysis methods see the Supplementary Materials. Figure 2b shows a representative data set from a thermomechanical experiment, which consists of axial strain and load as a function of time while the power dissipation is increased in a stepwise fashion. The strain vs. time response under Joule heating conditions shows strains that far exceed the room temperature elastic strain limit, indicating homogeneous deformation at elevated temperature in the nanowire. The initial strain of 3% (measured with respect to the initial state of the nanowire) represents the onset of near steady-state conditions where measurements were extracted; strains leading up to this point (not shown) were associated with settling of the power and load feedback circuits. Apparent transients are likely a convolution of both material effects and the experimental feedback control used to maintain constant force and power (see Supplementary Materials). Notably, the high strain to failure (>20%) indicates that Joule heating enabled superplastic-like deformation behavior in the amorphous state, which is indicative of high temperatures close to *T*_*g*_. The extent of deformation is further demonstrated in Fig. 2c, which shows the first micrograph and final micrograph before mechanical failure from the testing sequence. The white dashed lines illustrate the initial nanowire length. Notably, the observed steady-state strain-rate depends on the dissipated power (related to temperature); as the dissipated power increased the strain-rate correspondingly increased. During this experiment, the force data showed little deviation from the set point of 10 μN, demonstrating that the electrical current negligibly affected the applied force.

**Figure 3:**
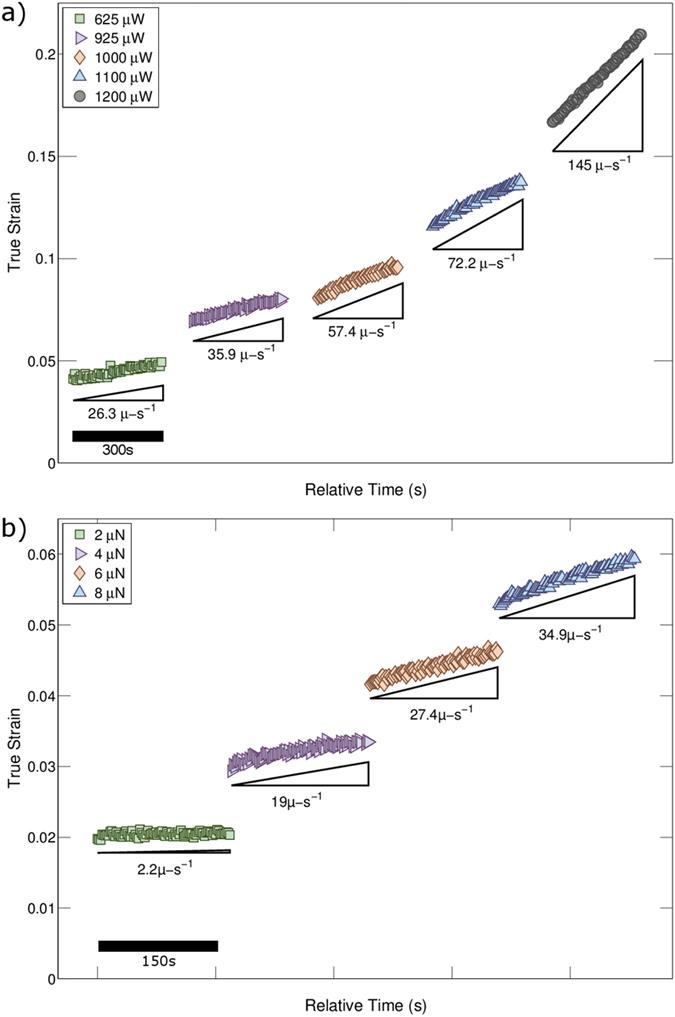
The strain data is converted to true-strain and representative regions of constant strain-rate and power dissipation are isolated as shown in (a). Here each segment is a period of steady state plastic deformation under an applied load of 10 μN. The strain-rate of each segment is indicated below the data. Potential stress dependent flow was examined by load-jump tests. A representative portion of strain data from a load jump test on a different nanowire specimen is shown in (**b**). In this example data 1.1 mW were dissipated in the nanowire and the force was increased in 2 μN increments from 2 μN to 8 μN.

From the strain-time data, segments of steady state plastic deformation were isolated. The true strain was determined from the measured engineering strain quantities based on the assumption of uniform deformation and constant volume during plastic deformation. Figure 3a shows the steady-state segments of true strain as a function of relative time extracted from the data shown in Fig. 2b. From these segments the steady-state strain-rates were determined as a function of power (~*T*; see Supplementary Materials), as indicated below each segment. When determining the strain-rate the average error was estimated to be less than 1% (see Supplemental Materials). To gain insight on the thermomechanical response, we performed additional load jump tests on individual nanowires. A portion of a load jump data set from a different nanowire specimen is shown in Fig. 3b. In this data segment, a constant power of 1.1 mW was applied to a single nanowire. Upon stabilization of power dissipation the force was increased quasi-instantaneously in 2 μN steps from 2 to 8 μN. After increasing the force a brief transient was observed followed by extended steady-state deformation. For all data sets the true stress was calculated from the area reduction given by the true strain. The uncertainty in stress was estimated to be 11% primarily from errors associated with determination of the cross sectional area. Using the true stress *σ* and true strain-rate 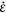, the viscosity was determined according to 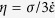 ref. 34. The resulting error in viscosity was estimated to be 11% across all tests. See Supplemental Materials for a discussion of the error estimation and analysis.

**Figure 4:**
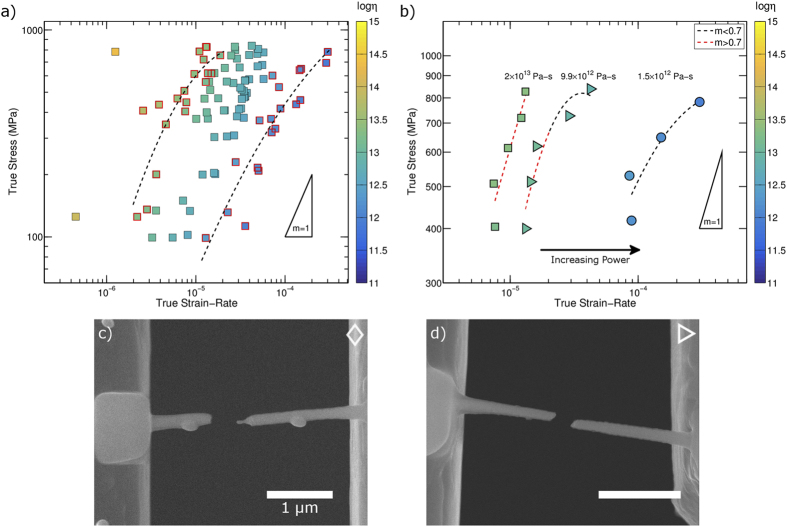
To account for potential stress effects on viscosity, the results are plotted as strain-rate versus stress in (a). The color scale indicates the log of viscosity. Here the results from all samples are plotted together. To emphasize possible non-Newtonian behavior the highest and lowest iso-viscosity points are highlighted. The dashed lines represent a polynomial fit to these points to guide the eye. A slope of 1, which indicates Newtonian behavior, is provided as a reference. To clarify the behavior observed in (**a**) we consider only samples subjected to load jump tests. A representative result from a load jump test on a single nanowire is provided in (**b**), as a function of increasing power (each symbol type indicates steady state at different power dissipation). Each segment is further labeled with its average viscosity. In addition, simple fits are shown to help approximate the strain-rate sensitivity, m. The slope of the simple fit is used as an approximated strain-rate sensitivity. The fits are colored according to the threshold used to determine the transition from Newtonian (*m* > 0.7) to non-Newtonian flow (m < 0.7). Micrographs showing the representative failure morphologies due to (**c**) necking down to a sharp point, denoted as a diamond, and (**d**) shear dominated failure, denoted as a triangle, are included. Such morphologies are indicative of Newtonian and non-Newtonian deformation respectively.

Performing tests over a wide range of applied stresses and powers enabled us to examine the full collection of thermomechanical testing results in terms of the steady-state strain-rate dependence on stress, as shown in Fig. 4a, where the symbol color indicates the estimated viscosity. Here the outlined data points show the highest and lowest approximate iso-viscosity lines, which can be compared to the constitutive law for Newtonian flow given above. A deviation from a strain-rate sensitivity of *m* = 1 would indicate non-Newtonian flow. The dashed lines are polynomial fits to the iso-viscosity points to guide the eye. From Fig. 4a it was observed that the stress varies sub-linearly with measured strain-rate, indicating a transition to non-Newtonian flow above a certain stress level.

Load jump tests provide additional insight into the transition from Newtonian to non-Newtonian behavior. Figure 4b shows the log-strain-rate vs. log-stress for a representative load jump test on a single nanowire, where each symbol type represents a steady-state condition at a different power dissipation. Additionally, each segment is labeled with the mean viscosity of the segment, which decreases as the dissipated power is increased. At each steady state condition, a polynomial function was fitted to log *σ* vs. 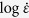 to determine the strain-rate sensitivity as a function of the experimentally measured strain-rate. To identify the transition from Newtonian to non-Newtonian flow, a threshold strain-rate sensitivity was selected. Strain-rate sensitivities greater than *m* = 0.7 were identified as Newtonian while strain-rate sensitivities less than *m* = 0.7 were labeled as non-Newtonian. In Fig. 4b, the polynomial fits are colored according to this threshold, with red indicating Newtonian (*m* > 0.7) and black indicating non-Newtonian (*m* < 0.7).

Changes in deformation morphology can be used as an additional signature of the transition to non-Newtonian deformation^30^, which we examine here to corroborate our thermomechanical measurements. While all samples demonstrated plastic deformation in excess of the elastic limit, the failure morphologies varied greatly as shown in Fig. 4c,d. Figure 4c shows failure by a drawing down to a sharp point and an absence of system spanning shear character. While such pronounced failure was only observed in one sample, similar morphologies were observed in many samples and signify Newtonian flow. Figure 4d presents a typical shear-dominated failure morphology, which is often associated with non-Newtonian deformation at elevated temperatures^30^.

Notably, transmission electron microscopy (TEM) observations of failure surfaces confirmed that the nanowires remained amorphous following thermomechanical testing. Indeed, TEM imaging revealed no observable crystallites within the deformed nanowires. Electron diffraction showed several diffuse scattering rings and no crystal spots, which is indicative of a disordered structure. In addition, from time-temperature-transition studies of this Pt-based glass, crystallization is not expected under these experimental conditions (for detailed analysis of potential crystallization in our nanowires see Supplementary Materials and Figure S3) until very long times, greater than 10 hours, as estimated from Legg *et al*.^35^. Therefore we conclude that our observations reflect the amorphous response and are not influenced by precipitation of crystallites.

**Figure 5:**
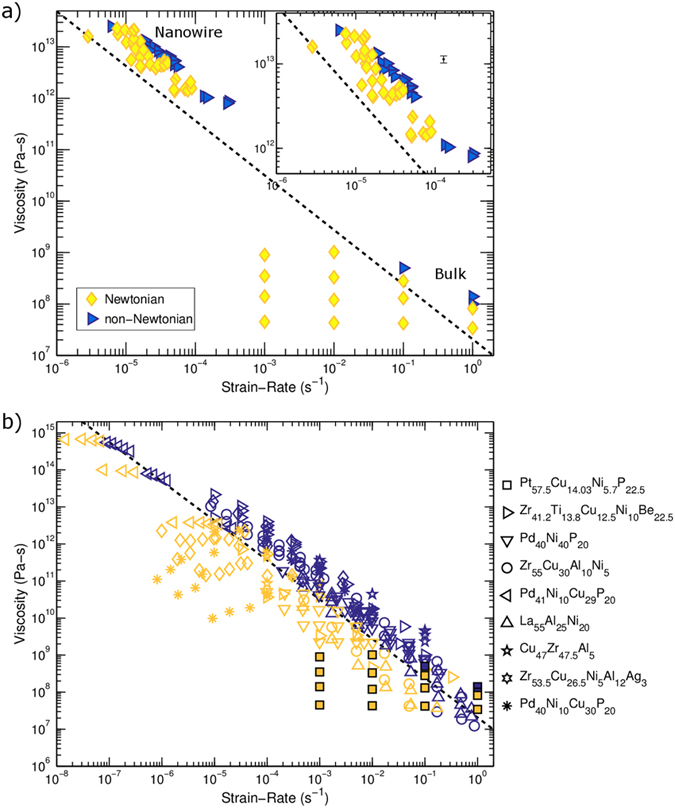
(**a**) A deformation map is constructed from observed failure morphologies (slashed symbols) from all tests and estimated strain-rate sensitivities determined from load jump tests on nanowires and bulk specimens. The inset highlights the nanowire results. From the deformation map a transition from Newtonian (yellow diamonds) to non-Newtonian deformation (blue triangles) is observed as the strain-rate increases. The symbol types match the deformation morphologies from Fig. 4c,d respectively. A black dashed line, determined from (**b**), is included to highlight the transition at different viscosities for compiled bulk data. Compiled bulk data from this work (filled squares) and the literature^26^,^27^,^28^,^29^,^36^,^37^,^38^,^39^,^40^ in (**b**) shows a deformation map over a range of viscosities and strain-rates. From the compiled data, an average transition from Newtonian to non-Newtonian deformation is determined as shown by the dashed black line. The black data marker in the inset represents the mean uncertainty in the determination of viscosity. The uncertainty in strain-rate is smaller than the marker size.

To characterize the complete spectrum of thermoplastic deformation in the MG nanowires a deformation map is constructed (Fig. 5a). The map is constructed from the observed failure morphologies (slashed symbols) and estimated strain-rate sensitivities from load jump tests. Here the symbol types correspond to the symbols from Fig. 4c,d. Failure morphologies showing no shear character or segments with high strain-rate sensitivities are described as Newtonian (diamonds). Failure morphologies showing significant shear character or segments with low strain-rate sensitivities are described as non-Newtonian (triangles). In cases when SEM observations were insufficient to definitively assign a failure category, TEM tilt series were used to corroborate SEM characterization. Regardless of the deformation type, extensive plastic deformation was observed before failure in all deformation regimes. From the deformation map a transition from Newtonian to non-Newtonian deformation is observed as the strain-rate is increased for a given viscosity. As the viscosity decreases, the transition shifts to higher strain-rates.

For a comparison across length scales over four orders of magnitude, included in Fig. 5a are results from compression of millimeter sized bulk specimens made from the same MG composition. Bulk experiments were conducted at higher temperatures to ensure good temperature control and avoid uncertainties with determination of temperature, resulting in lower viscosities. Consistent with the observation from nanowires, a transition from Newtonian to non-Newtonian deformation was observed. As the viscosity decreased the strain-rate required to undergo the transition increased.

To provide context to our results, flow data for nine bulk MGs obtained from literature was compiled in Fig. 5b 26,27,28,29,36,37,38,39,40. The deformation mode is again determined from the strain-rate sensitivity as previously described for the nanowire tension and bulk compression experiments presented here. Figure 5b shows that our measurements on the bulk Pt-based glass are largely consistent with other reports from the literature. Furthermore, the compiled data indicates the Newtonian to non-Newtonian transition to be consistent across the different glass compositions and over 8 orders of magnitude in viscosity. To highlight the transition, a dashed line is included in Fig. 5b. Critically, the transition from Newtonian to non-Newtonian deformation in bulk specimens occurs at similar strain-rates and viscosities regardless of glass composition. Thus we use the cumulative data set (including the Pt-based alloy used for nanowire measurements) for a large number of bulk metallic glass compositions to construct a linear fit to demarcate the transition between Newtonian and non-Newtonian flow, which is also included in Fig. 5a for direct comparison to the measured transitions in our Pt-based nanowires.

## Discussion

Using our results, we can examine the nature of thermoplastic flow in nanoscale specimens as compared to bulk specimens. Phenomenologically, nanoscale and bulk specimens behave similarly. Across all length scales a transition from Newtonian to non-Newtonian deformation is observed as the strain-rate increases. Failure morphologies also show similarity; when deforming nanowires in the Newtonian regime the failure surfaces show extensive deformation down to small diameters (Fig. 4c). In the non-Newtonian regime failure surfaces show shear character (Fig. 4d). These nanowire deformation morphologies agree with bulk observations^30^. The hot-pulling experiments further support this qualitative similarity between bulk and nanoscale response, as the estimated transition strain-rates are similar in magnitude to those of the bulk material and the deformation morphologies are consistent.

However, a direct comparison between nanoscale and bulk flow behavior indicates a measurable change in the onset strain-rate of non-Newtonian deformation at the nanoscale. In Fig. 5a, the average bulk transition, which does not depend on glass composition, determined from nine bulk MGs shown in Fig. 5b is reproduced as a black dashed line. When compared with the bulk transition, all nanowire observations, both Newtonian and non-Newtonian, are at higher strain-rates than the bulk flow transition. At a given viscosity, the transition strain-rate at the nanoscale increases nearly four-fold signifying a significant difference in the flow behavior in our nanowires. This increase is well above the estimated uncertainty in strain-rate of <1%. Similarly, at a given strain-rate the flow transition in nanoscale specimens occurs at viscosities nearly four times higher than in bulk. Again, the observed difference in the viscosity at which the Newtonian to non-Newtonian flow occurs in bulk versus nanoscale specimens is much greater than the estimated uncertainty in viscosity of 11%. See Supplemental Materials for a discussion of uncertainty estimation.

To explain the distinct behavior observed at the nanoscale we consider possible differences between nanowire and bulk specimens. Conceptually, the onset of non-Newtonian flow occurs when shear induced free volume generation surpasses thermal relaxation of free volume, resulting in a higher overall free volume and lower viscosities^33^,^41^. As the viscosity decreases the relaxation rate increases resulting in a new steady state at higher free volumes. From Fig. 5a it is observed that the nanowire specimens examined here maintain Newtonian flow at strain-rates up to four times greater than bulk MGs at a given viscosity. This suggests that the nanowire specimens may accommodate greater free volume generation before softening and establishing a new steady state. However, identifying the mechanism leading to the enhanced accommodation of free volume based on fitting bulk formalisms without an explicit temperature measurement is challenging. Instead we consider potential phenomenological explanations for the unique behavior observed at the nanoscale.

The flow behavior of the MG nanowires could be altered by both intrinsic and extrinsic factors. For instance the nanoscale dimensions of the specimen could result in an intrinsic alteration to the relaxation response due to the free surface. To accommodate increased free volume production during deformation the surface would need to act as a sink of free volume. In such a scenario, newly generated free volume would diffuse to and annihilate at the nanowire surface. This viewpoint is supported by molecular dynamics studies that specifically probe how free surfaces alter glass properties^42^,^43^,^44^. In all cases, the surface was observed to have enhanced free volume or atomic mobility as compared to the material core. Enhanced surface mobility has been demonstrated to lead to enhanced relaxation and increased ordering at simulated metallic glasses surfaces^45^. As a result, at reduced length scales the free surface may act as an effective sink of free volume. However, the extent of the surface is limited to the first several atomic layers potentially limiting the effectiveness of the surface at the sizes studied here.

Additionally, the fabrication methods, an extrinsic factor, could alter the free volume distribution relative to bulk structures and enable more free volume to be accommodated. Importantly, in the viscosity range examined relaxation times are still relatively long and the quenched-in free volume likely still influences the nature of deformation. During the molding process the nanowires are held at elevated temperature for an extended period of time resulting in structural relaxation and reduction of free volume. Such a mechanism is supported by Zhang *et al*. who demonstrated that free volume in nanowires is preparation dependent^44^. Wires produced by simulated hot imprinting showed lower free volume than the bulk as indicated by lower average Voronoi volumes. The reduction in free volume then manifested as distinctly different mechanical responses in agreement with experiments on MG nanowires, which illustrate a strong post-processing effects^46^,^47^,^48^. Thus the thermoplastic modeling technique employed to produce the nanowires studied likely resulted in a lower initial free volume, thereby enabling greater accommodation of free volume generated by plastic deformation.

Regardless of the mechanism resulting in the measured deviation from bulk behavior these results could be useful guides for future thermoplastic forming operations. The hot-pulling results demonstrate the scalability of thermoplastic forming and drawing. The sensitivity of the resulting structures to the strain-rate emphasizes the need for in depth studies of high temperature deformation at the nanoscale. Previous studies demonstrated deviations from bulk behavior, such as increasing viscosity at reduced length scales^19^ or nanoscale effects on crystallization^49^. Here we present the first instrumented study of high temperature flow in amorphous metallic nanowires. We observe that fundamentally the deformation mechanisms (i.e. the transition from Newtonian to non-Newtonian flow) remain unaltered at the nanoscale as indicated by changes in the strain-rate sensitivity and deformation morphology. However, measurements and observations of the transition from Newtonian to non-Newtonian flow suggest an increased accommodation of free volume at the nanoscale potentially allowing higher strain-rates or stresses to be employed during forming. Thus, while bulk measurements should serve as a suitable guide when designing a thermoplastic forming operation, this work provides important insights for thermoplastic forming of nanoscale features. Finally, our high temperature study presented here in conjunction with our previous studies at low temperature^46^,^47^ further indicate that the observed brittle-to-ductile transition^21^,^22^,^23^,^24^ in amorphous metals is likely mediated by processing effects which could be intertwined with true size effects.

## Conclusions

In summary, we presented a series of experiments to investigate the deformation of nanoscale MGs at elevated temperature. By deforming large arrays of nanowires it was observed that the deformation mode depended on the applied strain-rate. Initially long wires, leading to lower deformation rates, deformed in an apparently Newtonian fashion while initially short wires, with a higher relative strain-rate, deformed in a more non-Newtonian manner. While the hot-pulling experiments demonstrate the scalability of thermoplastic forming methods they also highlighted the need for in depth investigation of deformation at small sizes and elevated temperatures. To further investigate the high temperature deformation of nanoscale MGs we conducted thermomechanical tests on individual MG nanowires. By varying the power dissipated and applied load a deformation map was constructed from failure morphologies and estimated strain-rate sensitivities. The deformation map revealed a transition from Newtonian to non-Newtonian flow at strain-rates consistently higher than the average bulk transition. Shifting the flow transition to higher strain-rates is consistent with the accommodation of larger amounts of free volume produced by plastic deformation. We hypothesize that the difference in behavior is due to enhanced relaxation at the free surface and a lower steady state free volume resulting from the fabrication process. These experiments are expected to have potential impact on future thermoplastic forming operations by demonstrating that the deformation mechanisms are fundamentally similar across four orders of magnitude in length scale. The results of our work also suggest that higher strain-rates or stresses may be employed at the nanoscale owing to the enhanced capacity for free volume production in the Newtonian flow regime, and point to the importance of sample processing in mediating the ensuing deformation behavior.

## Experimental Methods

Large arrays of metallic glass nanowires were fabricated by hot-pulling from alumina nanotemplates (Fig. 1a)^50^. Pt_57.5_Cu_14.3_Ni_5.7_P_22.5_ metallic glass nanowires were thermoplastically formed at 270 °C between an alumina template and a microporous steel mesh (Step I). The microporous mesh and the nanotemplate were secured to the top and bottom heating plates, respectively. Subsequently, the top plate was cooled to 220 °C and slowly pulled while maintaining the bottom plate at 270 °C (Step II). This resulted in thermoplastic drawing of metallic glass nanowires with the variation in extent of elongation for different nanowires determined by the pore size distribution in the alumina template and the starting length of the nanowires formed during step I (Fig. 1b–e).

The thermomechanical behavior of individual metallic glass nanowires was measured *in situ* in the SEM by adapting our previously reported nanomechanical testing methods^46^,^47^. Briefly, Pt_57.5_Cu_14.3_Ni_5.7_P_22.5_ nanowires were produced by thermoplastic molding into a nanoporous alumina template at 270 °C at 130 MPa^6^. The template was subsequently removed by etching in KOH leaving freestanding nanowires. Individual nanowires were harvested *in situ* in the SEM using a nanomanipulator. The nanowires were mounted on a copper TEM half grid using Pt-containing electron beam induced deposition (EBID) material to ensure a strong mechanical mount and to allow electrical contact with the nanowire. The half grid was electrically isolated and mounted in a custom nanomechanical testing apparatus. The testing apparatus consisted of a six degree of freedom nanopositioning stage for sample alignment, a linear piezoelectric actuator to apply strains, and a MEMS based load cell with a load resolution of less than 20 nN, all coupled to external electronics via an electrical feedthrough. Nanowires were then attached to the load cell using Pt-EBID completing the load train and an electrical circuit. The experimental setup is illustrated schematically in Fig. 2a.

Thermomechanical experiments were conducted in creep-like conditions by applying a constant load on the sample while passing an electric current through the nanowire to resistively heat it. Custom software controlled the force by adjusting the position of the linear actuator; the standard deviation of the load during hold periods was approximately 90 nN. Current was supplied using a Keithley 2636A source-measure unit and sourced from the load cell, which was held at reference ground, to the TEM half grid, which was negatively biased, such that a constant power was dissipated in the nanowire. SEM image sequences were simultaneously captured and subsequently processed to calculate axial strains using digital image correlation techniques in which Pt-EBID fiducial markers were tracked throughout the image sequence^51^. Using this method, the plastic strain-rates (both transient and steady-state) were measured under a range of applied stresses and dissipated powers to determine the thermomechanical response of individual MG nanowires.

For comparison, bulk specimens of the same composition were compressed at elevated temperatures. Bulk ingots were cut into compression samples with diameter of 2 mm and height of 4 mm (aspect ratio 2:1). The samples were carefully polished to ensure parallelism. Uniaxial compression was subsequently conducted at comparable temperatures between 240 °C and 255 °C under strain-rates between 1 × 10^−3^ s^−1^ and 1 s^−1^.

## Additional Information

**How to cite this article**: Magagnosc, D. J. *et al*. Thermomechanical Behavior of Molded Metallic Glass Nanowires. *Sci. Rep*. **6**, 19530; doi: 10.1038/srep19530 (2016).

## Supplementary Material

Supplementary Information

## Figures and Tables

**Table 1:** Summary of large-scale nanowire hot-pulling parameters and resulting nanowire morphologies.

Bottom Plate Temperature	Top Plate Temperature	Pulling Speed, *v*	Average Initial Nanowire Length, *L*	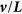	Deformation Morphology
270 °C	220 °C	10 μm/min	1.50 μm	0.11 s^−1^	Long nanowires with diameter reduction to ~50 nm
270 °C	220 °C	10 μm/min	0.45 μm	0.37 s^−1^	Sharp tips with little elongation
