# Supplementary material for: Thermomechanical Behavior of Molded Metallic Glass Nanowires
Source: Sci Rep. 2016 Jan 20;6:19530. doi: 10.1038/srep19530 (PMC4726219; doi:10.1038/srep19530)
Supplement: Supplementary Information [file srep19530-s1.pdf]

Supplemental Materials and Methods for:

THERMOMECHANICAL BEHAVIOR OF MOLDED METALLIC GLASS NANOWIRES

Daniel J. Magagnosc[1], Wen Chen[2], Golden Kumar[3], Jan Schroers[2], Daniel S. Gianola[1*]

[1] Department of Materials Science and Engineering, University of Pennsylvania, Philadelphia, Pennsylvania 19104, USA
[2] Department of Mechanical Engineering, Yale University, New Haven, Connecticut 06511, USA
[3] Department of Mechanical Engineering, Texas Tech University, Lubbock, Texas 79409, USA

[*] E-mail: gianola@seas.upenn.edu

Description of Thermomechanical Testing Methods and Analysis

Thermomechanical tests were performed using a custom built nanomechanical testing apparatus as previously reported[1,2]. The testing apparatus consists of a 3D nanopositioning stage (SmarAct GmbH), which enables closed-loop positioning over millimeter distances with nanometer precision, a linear piezoelectric actuator (Physik Instrumente), and a MEMS based load cell (FemtoTools AG) with a load resolution better than 20 nN. The load cells also enable electrical contact to the end of the load cell.

After manipulating nanowires to a TEM half grid using *in situ* pick-and-place methods in an SEM with a nanomanipulator (Kleindiek Nanotechnik GmbH), the conductive TEM grid electrically isolated from the testing apparatus by mounting to a custom PEEK sample holder. A compliant copper wire was used to electrically contact the TEM grid and hence the nanowire samples. Electrical feedthroughs allowed access to an external source measure unit (Keithely 2636A), which was controlled by a custom Labview program.

In situ creep tests were then conducted in the SEM by bonding the free end of a nanowire to the load cell with Pt-based EBID to complete the load train and electrical circuit. Constant load and constant power dissipation were maintained using proportional-integral-derivative

(PID) control. Over the course of a test the power dissipation and/or load were progressively increased while periodically capturing images in the SEM. When programming the PID controller, the feedback was intentionally slowed down in an effort to prevent mechanical or thermal instabilities. However such instabilities were still prevalent and often resulted in thermal run away and melting of the nanowire as shown in Figure S1a. After melting, the glass rapidly cools and can be found supported by a carbon sheath produced by imaging contamination or on the grips in the form of quenched spheres of metallic glass.

Strain was then calculated by tracking the positions of Pt-based EBID fiducial markers using digital image correlation techniques. After taking a sub image containing a single fiducial marker, a Gaussian to the average x and y profile as shown in Figure S1b. The marker coordinate is then determined as the peak position of the x and y profile fits. Tracking was accomplished by using the fit to the marker profile from the previous frame as the initial condition for fitting the marker profile in the current frame. The tracking resulted in a trajectory for each marker in both the x and y directions.

The trajectories were used to calculate the displacement of each marker and the average displacement of the two grips. The trajectories also allowed the potential in plane misalignment angle to be calculated for each frame. The true force along the nanowire axis was determined based on the resolved force and the potential in plane misalignment angle. In this way in plane angles were explicitly accounted for. However it is important to note that generally the angles were small and after accounting for the angle did not greatly affect the measurement. Critically, potential out of plane angles and displacements could not be accounted for and are a source of error.

Engineering strains were then calculated as the change in nanowire length and accounted for displacement of the grips along the load train and laterally due to thermal drift. The very first image of the testing sequence was used as the unstrained reference. Engineering stress was calculated by measuring the nanowire diameter before deformation and assuming a circular cross-section. Subsequently true stress and strain were calculated by assuming uniform deformation and constant volume during plastic deformation.

Scaling of Viscosity with Power Dissipation:

The viscosities determined from nine thermomechanical tests (each symbol type is a distinct nanowire) are shown in Figure S1 as a function of power dissipated times length squared (μW-μm^2), which normalizes to T-$T_{heat\ sink}$ from the solution to the heat equation for steady state heat generation and conduction[3]. The inset shows the measured viscosity as log-viscosity versus power dissipated times length squared. From Figure S1 it is observed that the measured viscosity varied from 2×10^{14} - 8×10^{11} Pa-s, which further indicates temperatures approaching T_g. Additionally, the shape of the viscosity-normalized power relation appears to be exponential. In glassy solids many relaxation processes vary exponentially with temperature, as often described by the Vogel-Fulcher-Tammann (VFT) relationship[4]. The VFT relation, which was derived from a reaction rate theory, describes the viscosity dependence on temperature by the fragility parameter. A low fragility, or strong glass, indicates a near-Arrhenius-like relationship between viscosity and temperature, whereas a high fragility (weak glass) indicates a strong deviation from Arrhenius dependence of viscosity on temperature. Thus the apparent exponential dependence of viscosity on normalized power indicates that the nanowire temperature is dependent on power dissipation, consistent with Joule heating. However, while the VFT parameters for the Pt-based

glass studied here can be estimated, the large scatter in the viscosity data precludes an accurate fit and a true conversion of dissipated power to nanowire temperature[5].

It is important to consider sources of scatter in the power dissipated. In particular the Pt-based electron beam induced deposition (EBID) material used as both mechanical grips and electrical contacts likely produces a large amount of scatter. Generally, the electrical properties of EBID are variable with a strong dependence on the deposition conditions[6]. However even at consistent deposition conditions larger scatter in resistivity is often observed[6–8]. Thus the large variability in the power dissipation and the corresponding material response is likely influenced by the EBID electrical contacts.

Preservation of Amorphous Structure

When assessing the flow behavior of a metallic glass it is critical to ensure that the response is not influence by nucleation and growth of crystalline domains. The bright field transmission electron micrograph in Figure 5d from the main text provides evidence that the nanowires remain fully amorphous. Here we provide additional evidence in through a selected area electron diffraction (SAED) pattern and additional bright field micrographs and calculations of expected crystallization times in Figure S2. The calculations of crystallization time were made following the results from Legg *et al.*[5] These calculations indicate that the nanowires could be held at temperatures near T_g for more than 10 hours before crystallization would become a concern. This calculation, in concert with the post-mortem TEM observations, provides significant confidence that crystallization is not an important factor when determining the flow response.

Estimation of Strain-Rate Sensitivity and Newtonian to non-Newtonian Transition

To estimate the strain-rate sensitivity $\left(m = {}^{\Delta \log \sigma}\!/_{\Delta \log \dot{\varepsilon}}\right)$ a polynomial function was first fitted to $\log \sigma$ versus $\log \dot{\varepsilon}$ for each nanowire specimen subject to load jump tests. The strain-rate sensitivity was then estimated by the derivative of the polynomial function evaluated at the measured strain-rate. The same process was employed to estimate the strain-rate sensitivity for all bulk data. This procedure is illustrated in Figure S3 using a subset of the data from Figure 4b.

The transition from Newtonian to non-Newtonian flow is determined using the estimated strain-rate sensitivities. When the strain-rate sensitivity is greater than 0.7 the flow is considered Newtonian; a strain-rate sensitivity less than 0.7 is described as non-Newtonian. Overall the location of the transition is insensitive to the chosen threshold. Choosing a different threshold will shift the boundary slightly. However since the same threshold was used for both the nanowire and bulk data the trends should be consistent regardless of the threshold value.

Estimation of Experimental Error

In this study several sources of error have been identified. The primary sources of measurement error originate from the measurement of MG nanowire diameter and cross-sectional area, noise in the load sensor, and the strain measurement by digital image correlation.

In this study, we have approximated the nanowire cross-section to be circular with a diameter, *d*. The diameter is taken as the average diameter measured at many locations over the length of the specimen. The typical variation in diameter is typically less than 10 nm, which is taken as an upper bound. We have not accounted for any evolution in cross-sectional shape; our conservative estimate on diameter may compensate for this uncertainty.

When performing digital image correlation the accuracy is determined by the pixel resolution of the SEM, the signal to noise ratio of the image, and the fitting of the fiducial marker

profiles. Here the strain noise floor is measured to be $\Delta\varepsilon \approx 2\times10^{-4}$ at 40,000x magnification. The measured noise in the force measurement during feedback control was $\Delta F \approx 90$ nN (200 Hz sampling rate). The error in strain-rate was determined as the root mean squared error determined during fitting of slope in strain versus time data within the steady state creep segments.

Finally for calculated quantities (i.e. stress and viscosity) we used the standard expressions for error propagation to estimate the experimental error[9]. Assuming all uncertainties are random and independent the uncertainty in a quantity, q, is described as

$$\frac{\Delta q}{q} = \sqrt{\left(\frac{\Delta r}{r}\right)^2 + \cdots + \left(\frac{\Delta w}{w}\right)^2 + \left(\frac{\Delta t}{t}\right)^2 + \cdots + \left(\frac{\Delta x}{x}\right)^2}.$$

Supplementary References:

1. Magagnosc, D. J. *et al.* Tunable Tensile Ductility in Metallic Glasses. *Sci. Rep.* **3,** 16–18 (2013).

2. Magagnosc, D. J. *et al.* Effect of ion irradiation on tensile ductility, strength and fictive temperature in metallic glass nanowires. *Acta Mater.* **74,** 165–182 (2014).

3. Gaskell, D. *An Introduction to Transport Phenomena In Materials Engineering, 2nd edition*. (Momentum Pr, 2012). doi:10.5643/9781606503577

4. Busch, R., Schroers, J. & Wang, W. H. Thermodynamics and Kinetics of Bulk Metallic Glass. *MRS Bull.* **32,** 620–623 (2011).

5. Legg, B. A., Schroers, J. & Busch, R. Thermodynamics, kinetics, and crystallization of Pt57.3Cu14.6Ni5.3P22.8 bulk metallic glass. *Acta Mater.* **55,** 1109–1116 (2007).

6. van Dorp, W. F. & Hagen, C. W. A critical literature review of focused electron beam induced deposition. *J. Appl. Phys.* **104,** 081301 (2008).

7. O'Regan, C. *et al.* Electrical properties of platinum interconnects deposited by electron beam induced deposition of the carbon-free precursor, Pt(PF3)4. *J. Vac. Sci. Technol. B Microelectron. Nanom. Struct.* **31,** 021807 (2013).

8. Dias, R. J. *et al.* Low resistivity Pt interconnects developed by electron beam assisted deposition using novel gas injector system. *J. Phys. Conf. Ser.* **371,** 012038 (2012).

9. Taylor, J. R. *An introduction to error analysis : the study of uncertainties in physical measurements*. (University Science Books, 1997).

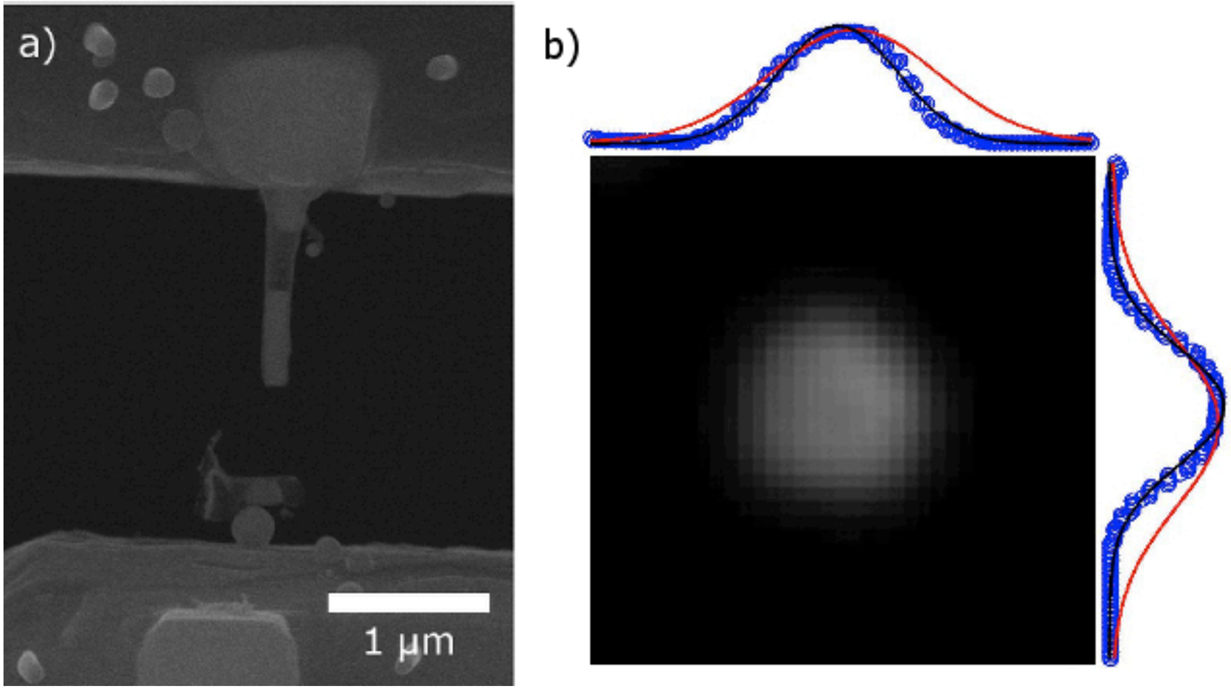

Figure S1: In a) an example of a melted nanowire is shown. Melting occurs due to mechanical and thermal instabilities. B) show a schematic of the fiducial marker tracking method. A sub image containing a single marker is shown. The x and y profiles are shown as blue circles. An initial guess fit is included in red and the profile fit is shown in black. The marker coordinate is then given by the center of the x and y profile fits.

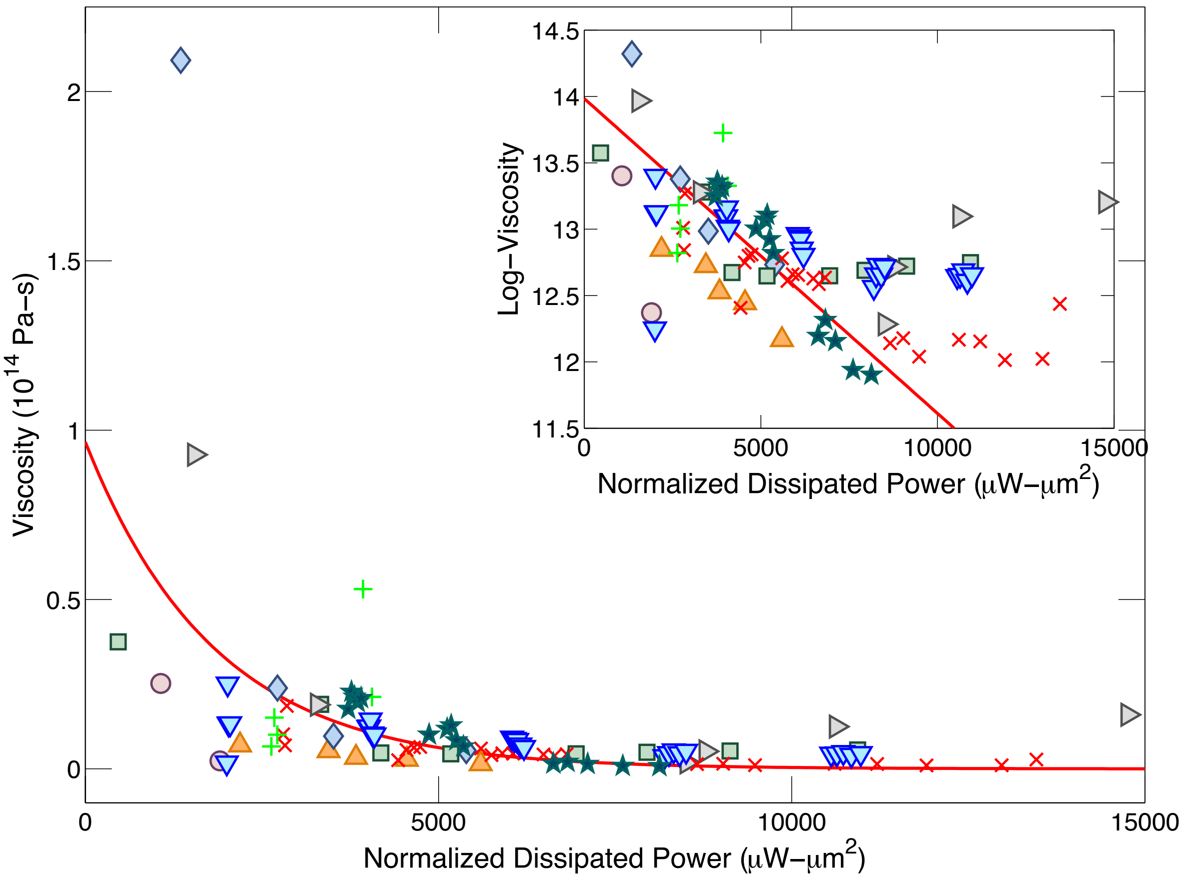

Figure S2: Using the measured strain-rates and stress the viscosity of the all thermoplastically deforming nanowires was determined. When plotted against power-length^2, the normalization for ΔT derived from the heat equation for conduction in 1 dimension, the viscosity shows an apparent exponential dependence. The inset shows the viscosity data as log-viscosity vs. power-length^2 to further show the apparent exponential relationship between viscosity and power-length^2.

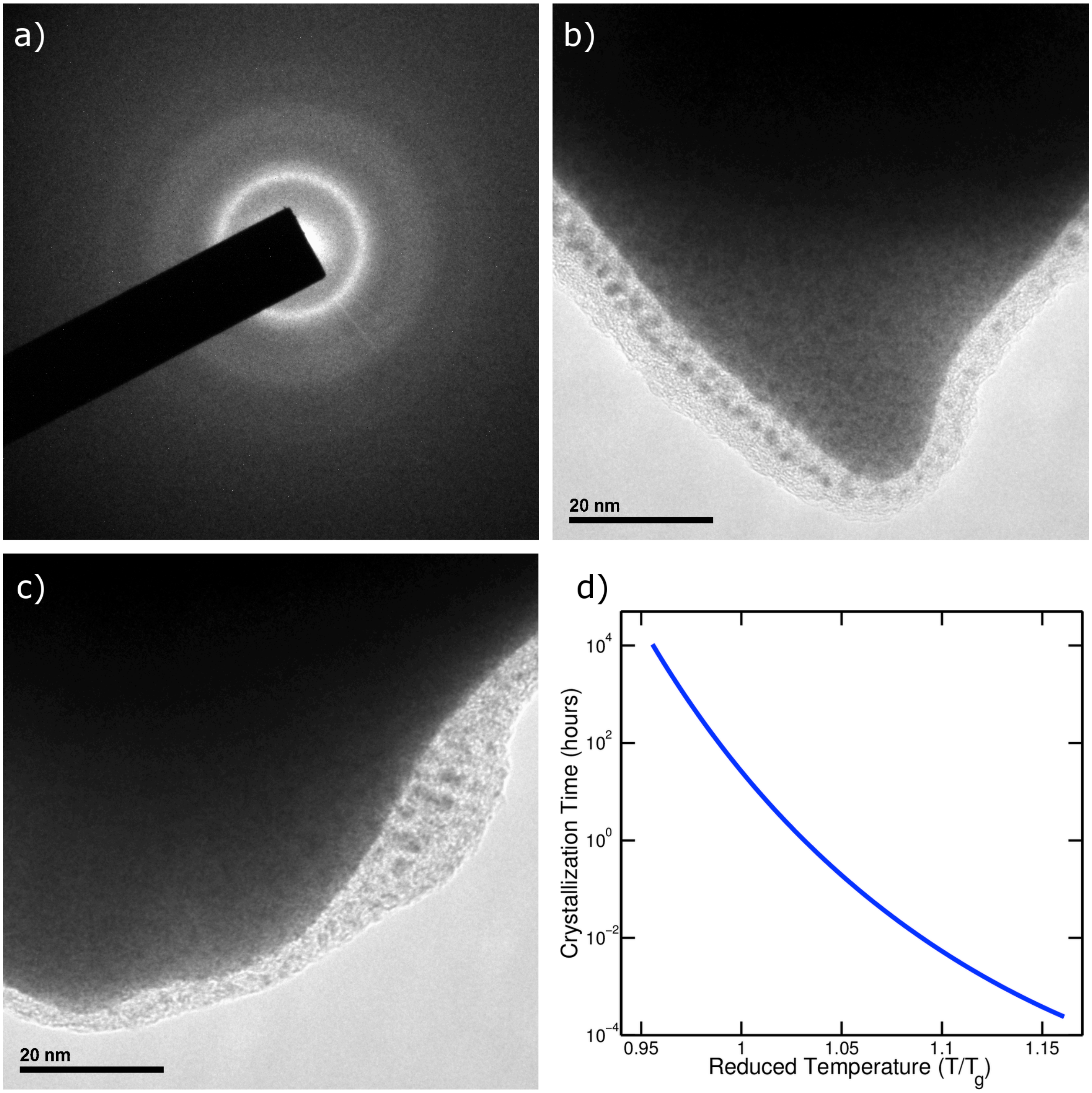

Figure S3: Post mortem TEM imaging provides evidence that the nanowires remain fully amorphous. a) SAED patterns taken on from a melted nanowire show no signs of crystal diffraction spots. Bright field images in b) and c) show uniform contrast and no lattice fringes within the nanowire failure surface. In d) the estimated time until the onset of crystallization as a function of temperature is calculated using parameters from [5].

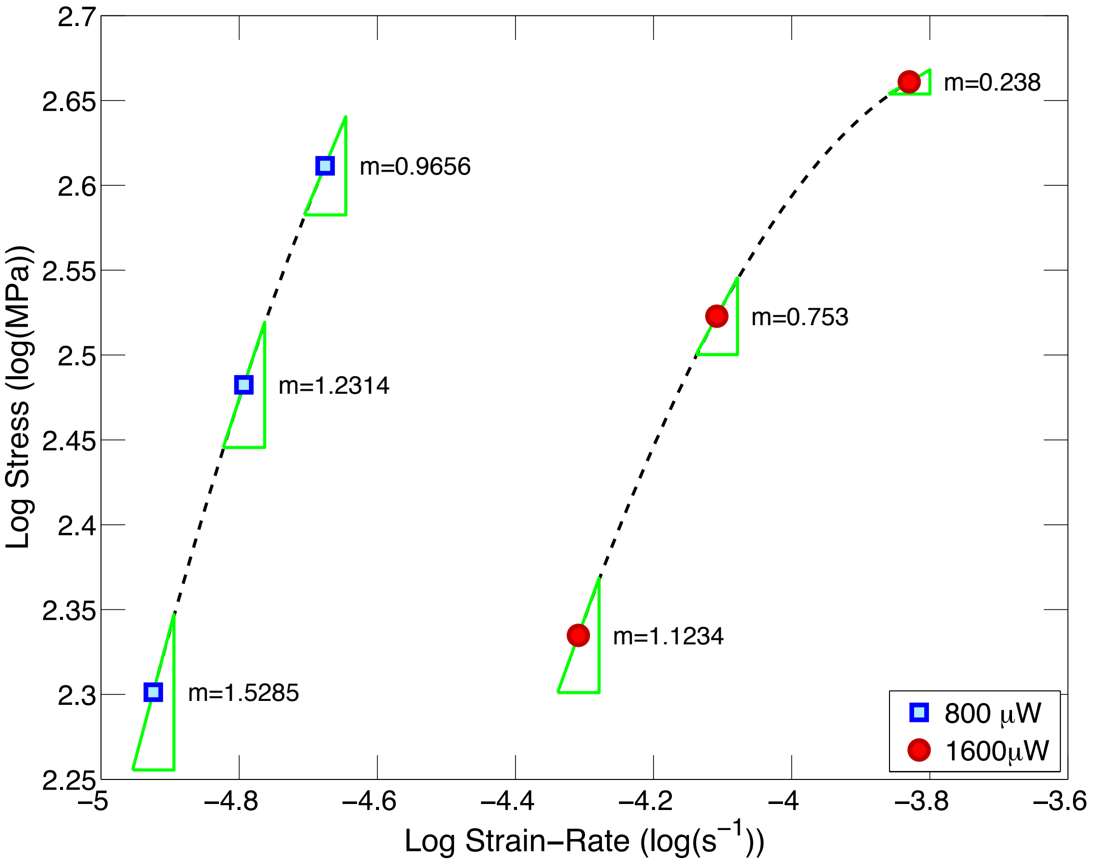

Figure S4: Strain-rate sensitivities are found by fitting load jump data with a polynomial function. By evaluating the derivative of the polynomial function at each data point the strain-rate sensitivity is estimated. The procedure is illustrated here a subset of the data from Figure 4b. The black dashed lines are the polynomial fit while the green triangles indicate the slope at each data point. Each triangle is labeled with its respective slope.
